# Bone Imaging of the Knee Using Short-Interval Delta Ultrashort Echo Time and Field Echo Imaging

**DOI:** 10.3390/jcm13164595

**Published:** 2024-08-06

**Authors:** Won C. Bae, Vadim Malis, Yuichi Yamashita, Anya Mesa, Diana Vucevic, Mitsue Miyazaki

**Affiliations:** 1 Department of Radiology, University of California—San Diego, San Diego, CA 92093, USA; wbae@health.ucsd.edu (W.C.B.);; 2 Canon Medical Systems Corporation, Otawara 324-0036, Japan

**Keywords:** MRI, FE, UTE, δUTE, osteoarthritis, bone fracture, image processing, CT

## Abstract

**Background:** Computed tomography (CT) is the preferred imaging modality for bone evaluation of the knee, while MRI of the bone is actively being developed. We present three techniques using short-interval delta ultrashort echo time (δUTE), field echo (FE), and FE with high resolution–deep learning reconstruction (HR–DLR) for direct bone MRI. **Methods:** Knees of healthy volunteers (*n* = 5, 3 females, 38 ± 17.2 years old) were imaged. CT-like images were generated by averaging images from multiple echoes and inverting. The bone signal-to-noise ratio (SNR) and contrast-to-noise ratio (CNR) were determined. **Results:** The δUTE depicted a cortical bone with high signal intensity but could not resolve trabeculae. In contrast, both the FE and FE HR–DLR images depicted cortical and trabecular bone with high signal. Quantitatively, while δUTE had a good bone SNR of ~100 and CNR of ~40 for the cortical bone, the SNR for the FE HR–DLR was significantly higher (*p* < 0.05), at over 400, and CNR at over 200. **Conclusions:** For 3D rendering of the bone surfaces, the δUTE provided better image contrast and separation of bone from ligaments and tendons than the FE sequences. While there still is no MRI technique that provides a perfect CT-like contrast, continued advancement of MRI techniques may provide benefits for specific use cases.

## 1. Introduction

Knee osteoarthritis (OA) represents one of the most common forms of arthritis, affecting millions of individuals worldwide and serving as a major cause of disability, particularly in the aging population. During OA, morphologic changes to the bone such as osteophyte formation (bony projections that develop along the edges of bones) may occur [1]. Bone fractures within the knee are not uncommon in the context of OA, adding a layer of complexity to its management [2]. Magnetic resonance imaging (MRI) has become the modality of choice for the assessment of knee OA by allowing detailed visualization of joint structures in a non-invasive manner. In particular, sequences have been developed to evaluate the bone changes associated with OA, including ultrashort echo time (UTE) [3,4] and zero echo time (ZTE) [3,5]. These advanced imaging techniques have the unique capability to capture signals from short T2 < 1 ms in tissues like the bone, which are typically invisible on conventional MRI sequences, such as spin echo, which utilizes TEs longer than ~10 ms. UTE and ZTE MR sequences have been used increasingly for bone-imaging applications [3,5,6,7,8]. Despite their advantages, UTE and ZTE MRI are not without shortcomings; they often suffer from low signal-to-noise ratios and resolution [9], which may lead to diagnostic uncertainty. Additionally, the complexity of UTE and ZTE sequences often requires longer acquisition times and specialized equipment, which may not be readily available in all clinical settings. The need for advanced technical expertise to optimize and interpret these images further complicates their routine clinical use. There are other UTE-like MR techniques that have been developed, such as 3D, fast, large-angle spin echo (FLASE) [10] and sweep imaging with Fourier transform (SWIFT) [11,12], but these have not seen a widespread clinical adoption.

To address these limitations from different angles, there has been a growing interest in the development of additional MRI techniques that can offer more precise and reliable imaging of cortical and trabecular bones of the knee. Among these, short-interval multi-echo delta UTE (δUTE) [13] has been introduced for precise T2* measurements, along with UTE applications in lung imaging [14,15], utilizing very short TE intervals (~0.4 ms) to acquire multiple short TE images with good bone contrast. While this is similar to multi-echo UTE [16,17], the utilization of shorter echo spacing may provide better contrast for bone and less susceptibility to artifact for thinner trabecular bone. Another technique is fast field echo [18] or spoiled gradient echo [19] acquisition, with the advantage of these sequences being their wide availability from most vendors, field strength, and high spatial resolution. In addition to the acquisition, post-processing is important for providing good bone contrast and CT-like appearance. Conventional image processing such as rescaled echo subtraction [20], simple inversion of a single echo [19], or summation of all but the last echo followed by the last echo [18] have been attempted with varying degrees of success. Additionally, recent developments in high-resolution deep learning reconstruction (HR–DLR) algorithms [21] may improve the source images by lowering noise and increasing spatial resolution, which are important benefits for clinical evaluation of the bone. HR–DLR also has the potential to reduce scan time.

The purpose of this study was to describe and compare several MRI techniques—short-interval multi-echo delta UTE (δUTE) and multi-echo 3D FE—with a particular focus on their application with and without DLR. The long-term goal is to establish improved methods for visualizing bone changes in knee OA, potentially leading to enhanced diagnostic capabilities and better patient outcomes. By achieving CT-like contrast through simple post-processing steps, these techniques may set a new standard for bone imaging in the evaluation of knee OA.

## 2. Materials and Methods

Study Design: This is a prospective cross-sectional study to compare the imaging performance between three different MRI sequences for imaging bones in the human knee.

Subjects: For this study of technical comparison, we recruited five healthy volunteers without symptoms of knee pain. There were 3 females and 2 males, with an average (standard deviation) age of 38 years old (17.2 years old). Only the right-leg knees of each individual were imaged.

**Figure 1:**
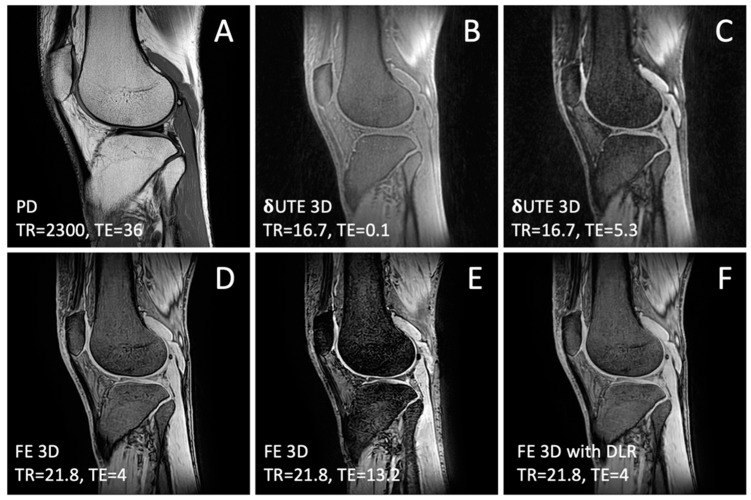
Source MRI images of (**A**) proton density (PD) fast spin echo, (**B**,**C**) delta ultrashort echo time (δUTE) at varying echo times (TE), (**D**,**E**) field echo (FE) at varying TE, and (**F**) FE with deep learning reconstruction (DLR).

MRI: The volunteers were imaged in a 3-Tesla MRI scanner (Galan, Canon Medical Systems Corp., Otawara, Japan) fitted with a clinical transmit/receive knee coil. The imaging plane was sagittal. One routine anatomical sequence and two bone-oriented sequences were used to scan each subject. The routine anatomical sequence was a fast spin echo T2 weighted fat suppressed with the following scan parameters: time to repeat (TR) = 2300 milliseconds (ms), time to echo (TE) = 36 ms, field of view (FOV) = 160 mm (mm), image matrix size = 448 × 448, slice thickness = 3 mm, and echo train length (ETL) = 7. For bone imaging, the sequences were as follows: (1) 3D δUTE multi-echo sequence [13] with the following parameters: TR = 16.7 ms; TE = 0.096, 0.5, 0.9, 1.3, 2.7, 5.3 ms; FOV = 200 ms; matrix = 256 × 256; slice = 1 mm and (2) 3D field echo (FE) multi-echo sequence with parameters of the following: TR = 21.8 ms; TE = 4, 8.6, 13.2, 17.8 ms; FOV = 200 mm; matrix = 288 × 288; and slice =1 mm. Lastly, the 3D FE images were post-processed on the scanner console using a built-in high resolution deep learning reconstruction (HR–DLR [21]) version of the FE image (FE HR–DLR), resulting in intensity correction and super-resolution of the image matrix to 864 × 864. Briefly, HR–DLR uses a deep convolutional neural network [22] that was trained on high resolution and high signal-to-noise ratio images for the purpose of enhancing lower quality input images with low resolution and high noise [21]. These source images are shown in Figure 1.

**Figure 2:**
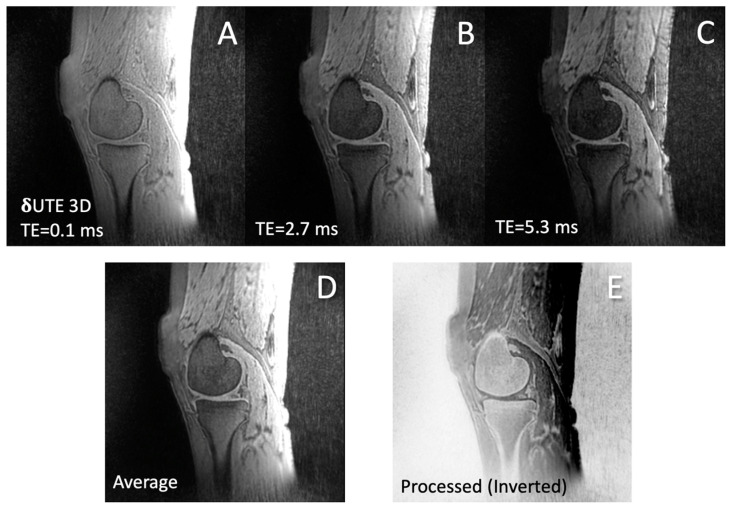
Image processing procedure. Source images from varying TEs (**A**–**C**) were first averaged (**D**) and then inverted (**E**) to create the final processed image with CT-like contrast for bone.

**Figure 3:**
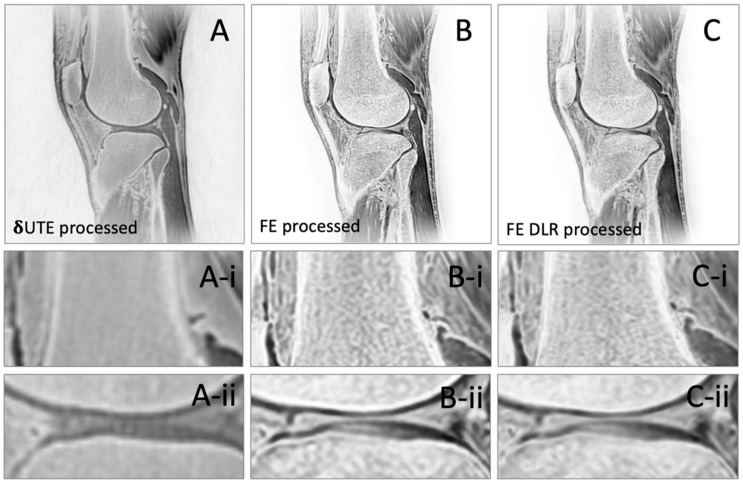
Representative processed images with CT-like contrast, created from (**A**) δUTE, (**B**) FE, and (**C**) FE HR–DLR source images. Images below show magnified sections of (**i**) cortical bone and (**ii**) tibiofemoral contact region.

Image Processing: The goal of the image processing was to obtain a CT-like appearance of the bone from the MRI images acquired with the δUTE, FE, and FE HR–DLR sequences. A simple processing was performed directly on the scanner console. For each sequence, all of the source images from different TEs (Figure 2A–C) were averaged (Figure 2D) and then inverted to yield the final processed image (Figure 2E), following Equation (1).

(1) $$Processed\;Image=\frac{n}{\sum_{i=1}^{n}{Source\;Image}_{i}}$$

where *i* = source images corresponding to each TE, and *n* = number of TEs. The intention for the averaging was to reduce the noise, and the inversion was to give a high signal intensity to the bone. Representative processed images from all three sequences are shown in Figure 3.

**Figure 4:**
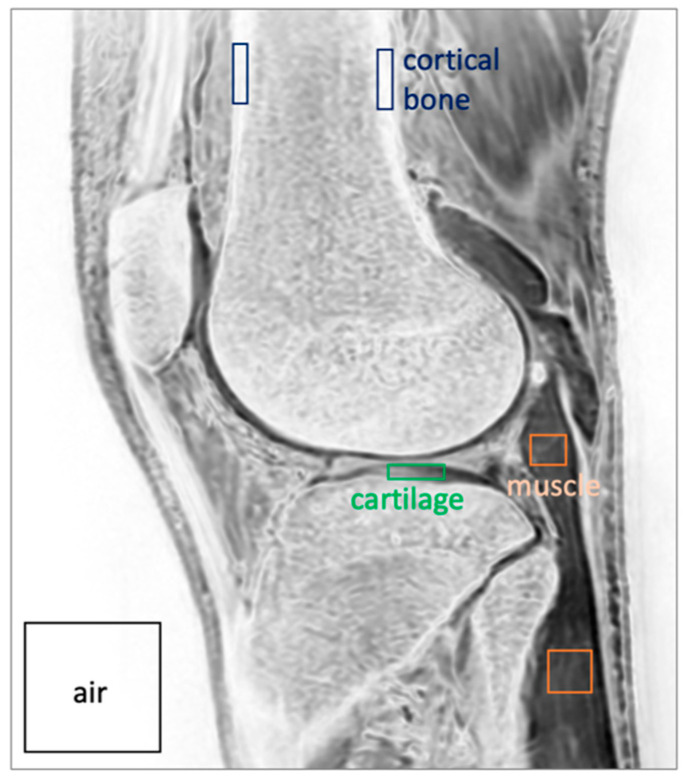
Regions of interest including cortical bone, muscle, cartilage, and air, analyzed to determine SNR and CNR.

Signal-to-Noise Ratio (SNR) and Contrast-to-Noise Ratio (CNR): Regions of interest (ROIs) were drawn on a selected slice of the processed images through the lateral compartment of the knee for cortical bone, muscle, and cartilage (Figure 4) to determine the mean signal intensity (*SI_mean_*) for each ROI. The ROI was also placed in the background (air) to determine the standard deviation (*SI_SD_*) of the noise signal intensity.

Using these, the SNR was determined as the mean signal intensity divided by standard deviation of the noise in the air [23,24] (Equation (2)).

(2) $${SNR}^{1}=\frac{{SI}_{mean}^{1}}{{SI}_{SD}^{noise}}$$

CNR was determined as the difference in mean signal intensity between selected ROIs divided by standard deviation of the noise [24,25] (Equation (3)).

(3) $${CNR}^{1-2}=\frac{{SI}_{mean}^{1}{-SI}_{mean}^{2}}{{SI}_{SD}^{noise}}$$

Statistics: The values between the MRI techniques tailored for bone (δUTE, FE, and FE HR–DLR) were compared using ANOVA with a post hoc Tukey’s test [26,27], using Systat statistical analysis software (v12, Grafiti LLC, Palo Alto, CA, USA). The significance level was set at 5%.

**Figure 5:**
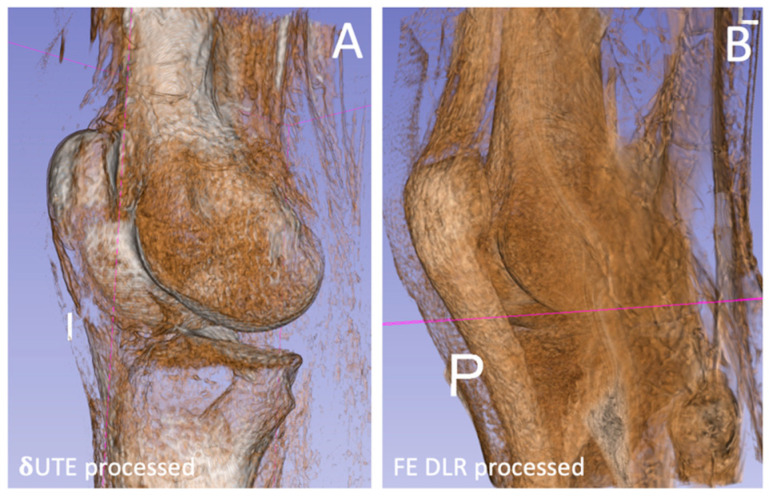
Three-dimensional bone renders created from (**A**) δUTE and (**B**) FE DLR data shows marked differences in appearance and the types of tissues being rendered.

3D Rendering: On the processed images, the bright background was segmented and inverted to low signal using a region-growing technique on ITK–SNAP [28]. This was performed in Active Contour 3D Segment Mode with threshold pre-segmentation and placing several seed points on the background, followed by region-growing. Once the background was inverted, the images were 3D-rendered using 3D Slicer software [29] using manual thresholding to best visualize the bone (Figure 5).

## 3. Results

Source Images: Compared to the conventional T2 image (Figure 1A), the δUTE images (Figure 1B,C) depicted the bones with a low signal intensity and most other tissues with a high signal intensity. The FE images (Figure 1D,E) also depicted the bone with a low signal but a few other tissues (ligaments and tendons) with low signal intensity. The FE HR–DLR (Figure 1F) provided a higher resolution, lower noise, and flatter intensity field compared to the source FE image (Figure 1D).

CT-like Images: After processing, the δUTE images (Figure 3A) had a CT-like contrast, depicting cortical bone with high signal intensity (Figure 3A-i) but most other soft tissues with mid-to-low signal intensity. However, the δUTE-processed images could not resolve trabecular bone (Figure 3A-ii). In contrast, both the FE- (Figure 3B) and FE HR–DLR-processed images (Figure 3C) depicted the cortical and trabecular bone with high signal intensity. While the FE and FE HR–DLR images were similar overall, the FE HR–DLR appeared slightly sharper (Figure 3C-i) and depicted cortical bone as thinner structure (Figure 3C-i,ii), while the FE images (Figure 3B) appeared more contrasted particularly between the trabecular bone and the bone marrow. This suggested that, due to higher resolution, the FE HR–DLR could be useful for evaluating small bone injuries, such as hairline fracture, or changes in the trabecular bone, for example, during osteoporosis.

SNR and CNR: Table 1 summarizes the SNR and CNR values for the processed CT-like images. While the δUTE-processed images had a good SNR ~100 and CNR ~40 for the cortical bone, the values were generally greater when measured on the FE and FE HR–DLR. The bone SNR for the FE HR–DLR averaged over 400, with the bone–muscle CNR being over 200. There was a trend (*p* < 0.1) of higher differences in the bone SNR and cartilage SNR between the δUTE and FE HR–DLR.

3D Bone Rendering: Figure 5 shows the 3D-rendered images with the threshold set manually to render the bone, comparing the δUTE (Figure 5A) and FE HR–DLR (Figure 5B) data. While neither could perfectly segment the bone based on the signal intensity alone, the δUTE fared somewhat better, unlike the FE HR–DLR, which rendered many non-bone tissues with a similarly high signal intensity as bone tissues (e.g., tendons and ligaments).

## 4. Discussion

The advancements in MRI techniques that deliver CT-like appearance for the evaluation of bone structure are driven by the clinical need for high-resolution and accurate assessments of bone integrity, useful for the evaluation of bone fracture and loss or remodeling of the trabeculae. Our findings are encouraging, suggesting that these MRI sequences, coupled with simple image processing, can indeed approximate the general appearance of the CT images, albeit with limitations.

Our study compared short-interval multi-echo delta UTE (δUTE) and multi-echo 3D FE sequences and demonstrated that while both sequences can depict cortical bone well, the FE technique provided greater SNR and CNR for cortical bone, and in addition, depicted finer details of the trabecular bone structure (Figure 3B). Additional enhancement from the use of HR–DLR was very subtle yet provided significant improvements in the SNR and CNR values and a visibly sharper (i.e., thinner) depiction of the cortical bone. Conversely, for 3D rendering of the bone surface, the δUTE technique seemed superior, suggesting that the best choice of technique might depend on the specific need, such as fracture detection, where a high spatial resolution is preferred, vs. pre-surgical planning, where the overall shape of the bone is of interest. Note that regular multi-echo UTE has the first and second TE intervals at 2.2 ms, whereas the δUTE allows for collecting a shorter TE interval (i.e., 0.4 ms). Collecting TEs between 0.096 ms and 2.2 ms using δUTE may contribute to a more bone-like imaging.

Our study builds upon recent reports that utilized similar approaches, which acquired source images using conventional fast field echo [18], gradient echo [30], or spoiled gradient echo [19], followed by post-processing to yield CT-like images. These studies have demonstrated that these conventional sequences can indeed provide a good depiction of the cortical outline of the bones, and in one study [19], high sensitivity, specificity, and accuracy (all above 90%) for detecting the bone fracture was demonstrated. Our result generally corroborated these past results in terms of improving visualization, and additionally provided quantitative SNR and CNR values that showed a trend of higher SNR for the FE HR–DLR-processed images compared to δUTE-processed images, which in itself provides improved bone imaging compared to conventional MRI [19,30,31].

This is an early study with many limitations. While the feasibility of healthy volunteers has been shown, a comparative study using CT reference images is needed. Past studies have shown that MRI, if specialized for bone imaging, can have good geometric accuracy [32,33,34] and similar diagnostic performance [35] against CT. Clinical patients or specimens with osseous injuries (e.g., fracture) or pathologies (e.g., osteophyte) would be helpful for determining the clinical reliability of MR-based bone imaging techniques including ours. As an early development, only a small number of subjects were used, but the differences in SNR and CNR appear substantial and the trend likely will be similar with greater numbers. In addition, the image-processing technique (i.e., average images from multiple echoes then invert) was chosen for simplicity and to reduce noise; we have yet to explore the finer details, such as how the inclusion of only certain images or using a different image calculation would affect the results of our study.

It should be acknowledged that, despite the substantial progress, the search for an MRI technique that provides a universally CT-like contrast continues. Each method brings its own set of advantages and trade-offs in terms of image quality, resolution, and practicality in a clinical setting. Thus, the selection of an appropriate imaging technique should be tailored to the particular diagnostic or research need. A promising avenue not yet discussed includes the use of artificial intelligence to synthesize CT-like images from MRI images [6,34,36]. This approach appears to yield the closest resemblance in terms of tissue contrast; however, the results will be both input (i.e., MR sequence used for the source image, anatomy, pathology, etc.) and deep learning model-dependent, and that is currently universally on all MR images.

## 5. Conclusions

In conclusion, while MRI has not yet fully matched the bone-imaging capabilities of CT, the ongoing refinement of techniques including δUTE and FE, particularly with HR–DLR, offers promises for achieving this goal. This study shows that current MRI techniques are able to image bone with high contrast, but this study lacks specificity for bone, requiring the reader to have a prior knowledge of knee anatomy in order to evaluate it properly. While the long-term goal of completely replacing CT with MRI is yet to be realized, future research and technological development will undoubtedly push the boundaries, and MRI might one day provide a one-stop imaging solution for bone imaging.

## Figures and Tables

**Table 1:** Mean and standard deviation of SNR and CNR values from various regions of interest.

	Mean (+/−Std. Dev.) Values for Each Sequence			ANOVA
Measurement	δUTE processed	FE processed	FE HR-DLR processed	*p*-value
Bone SNR	104 (19.3)	304 (271)	410 (179)	0.086
Muscle SNR	63.1 (22.2)	116 (70.0)	168 (64.4)	0.716
Cartilage SNR	69.8 (23.5)	166 (141)	233 (96.1)	0.067
Bone-Muscle CNR	40.5 (8.4)	187 (205)	242 (139)	0.137
Bone-Cart CNR	33.8 (6.6)	138 (148)	177 (103)	0.124

## Data Availability

The data that support the findings of this study are not publicly available due to reasons of sensitivity. Anonymized data may be available from the corresponding author upon the review of the request. Data are located in controlled access data storage at the corresponding author’s institution.
